# Formulation of a Thermosensitive Imaging Hydrogel for Topical Application and Rapid Visualization of Tumor Margins in the Surgical Cavity

**DOI:** 10.3390/cancers14143459

**Published:** 2022-07-16

**Authors:** Ethan Walker, Daan G. J. Linders, Eric Abenojar, Xinning Wang, Hans Marten Hazelbag, Marieke E. Straver, Okker D. Bijlstra, Taryn L. March, Alexander L. Vahrmeijer, Agata Exner, Matthew Bogyo, James P. Basilion, Brian Straight

**Affiliations:** 1 Department of Biomedical Engineering, Case Western Reserve University, Cleveland, OH 44106, USA; yvv@case.edu (E.W.); xinning.wang@case.edu (X.W.); agata.exner@case.edu (A.E.); jxb206@case.edu (J.P.B.); 2 Department of Surgery, Leiden University Medical Center, 2333 ZA Leiden, The Netherlands; d.g.j.linders@lumc.nl (D.G.J.L.); o.d.bijlstra@lumc.nl (O.D.B.); a.l.vahrmeijer@lumc.nl (A.L.V.); 3 Department of Radiology, Case Western Reserve University, Cleveland, OH 44106, USA; eca20@case.edu; 4 Department of Pathology, Haaglanden Medical Center, 2512 VA The Hague, The Netherlands; h.hazelbag@haaglandenmc.nl; 5 Department of Surgery, Haaglanden Medical Center, 2512 VA The Hague, The Netherlands; m.straver@haaglandenmc.nl; 6 Department of Clinical Pharmacy and Toxicology, Leiden University Medical Center, 2333 ZA Leiden, The Netherlands; t.l.march@lumc.nl; 7 Department of Pathology, Stanford University, Stanford, CA 94305, USA; mbogyo@stanford.edu; 8 Department of Microbiology and Immunology, Stanford University, Stanford, CA 94305, USA; 9 Akrotome Imaging Inc., Charlotte, NC 28205, USA

**Keywords:** human breast cancer, optical imaging, tumor margins, surgical cavity

## Abstract

**Simple Summary:**

We have developed a formulation for an innovative, quenched, cathepsin-targeted, fluorescent molecular probe to enhance resection quality for several solid-tumor cancers. Unlike other formulations for imaging probes or tracers in development and entering the clinic, which require systemic administration hours before the procedure, this current formulation is applied topically into the surgical cavity immediately after a standard of care resection. Within minutes of application, the probe activates in the presence of residual cancer in the surgical wound and provides a strong fluorescent signal that precisely delineates any remaining cancer, enabling a more complete resection. Utilization of this imaging gel formulation for topical application to detect breast cancer in the surgical cavity during surgery has the potential to reduce re-excisions, with consequent savings in healthcare costs and enhancement in patient quality of life.

**Abstract:**

Background: Tumor-positive surgical margins during primary breast cancer (BCa) surgery are associated with a two-fold increase in the risk of local recurrence when compared with tumor-negative margins. Pathological microscopic evaluation of the samples only assesses about 1/10 of 1% of the entire volume of the removed BCa specimens, leading to margin under-sampling and potential local recurrence in patients with pathologically clean margins, i.e., false negative margins. In the case of tumor-positive margins, patients need to undergo re-excision and/or radiation therapy, resulting in increases in complications, morbidity, and healthcare costs. Development of a simple real-time imaging technique to identify residual BCa in the surgical cavity rapidly and precisely could significantly improve the quality of care. Methods: A small-molecule, fluorescently quenched protease-substrate probe, AKRO-QC-ICG, was tested as part of a thermosensitive imaging gel formulated for topical application and imaging of the BCa surgical cavity. Results: More than forty formulations of gel mixtures were investigated to enable easy fluid application and subsequent solidification once applied, preventing dripping and pooling in the surgical cavity. The final formulation was tested using human BCa orthotopic implants in nude and NSG patient-derived xenografts (PDX) mice. This formulation of Pluronic F-127/DMSO/AKRO-QC-ICG imaging gel was found to be a good solvent for the probe, with a desirable thermo-reversible solid–gel transition and mechanical strength for distribution of AKRO-QC-ICG on the surfaces of tissue. It demonstrated excellent ability to detect BCa tissue after 10 min exposure, with a high signal-to-noise ratio both in mouse xenografts and freshly excised human lumpectomy tissue. The in vivo efficacy of the AKRO-QC-ICG imaging gel to detect BCa revealed the levels of sensitivity/specificity = 0.92/1 in 12 nude mice, which was corroborated with the sensitivity/specificity = 0.94/1 in 10 PDX mice. Conclusions: Utilization of Pluronic F-127/DMSO/AKRO-QC-ICG imaging gel for topical application to detect BCa in the surgical cavity during surgery has the potential to reduce re-excisions, with consequent savings in healthcare costs and enhancement in patient quality of life.

## 1. Introduction

For 2022, breastcancer.org projects 287,850 new cases of invasive breast cancer (BCa) and 51,400 new cases of non-invasive (in situ) BCa in women. Surgical removal of the primary BCa with or without systemic therapy is the standard of care for this disease. Since the observed mortality rates after breast conserving surgery (BCS) are equivalent to those after mastectomy, nearly 75% of US BCa surgeries are currently BCS. Approximately 60% of patients with Stage I–III disease choose this option, with 16% of those having Stage III disease [1,2].

The microscopic status of excision margins is critically important in BCS (lumpectomy) [3]. “Positive surgical margin” is a pathology term indicating that invasive carcinoma or ductal carcinoma in situ (DCIS) is touching a tissue edge of a lumpectomy specimen (current guidance recommendations; see also Ref. [4]. Among patients treated by BCS and radiation therapy, positive margins are associated with a two-fold increase in the risk of local recurrence when compared with negative margins [5,6]. The key element of determination of margin status is a pathological exam of the lumpectomy samples. Following surgery, the excised specimen is typically marked with ink to provide orientation, cut into 2–3-mm-thick portions, fixed, paraffin embedded, and one (or more) 3–5-μm-thick section is cut from each of these portions for histological analysis. The margins on each section are then microscopically examined by a pathologist days after surgery is completed and the patient has been discharged from the hospital.

Despite the development of several technologies to assay tumor margin infiltrates during the surgical procedure, e.g., cytology and frozen sections, none have proven robust enough to gain widespread clinical acceptance. A recent survey revealed that only 48% of 351 American surgeons grossly examined margins intra-operatively with a pathologist and even fewer used frozen sections or imprint cytology [7]. Because pathological analysis of BCS specimens takes 3–5 days, patients must be called back to the hospital to undergo secondary surgeries, creating an enormous burden in time, cost, and patient anxiety. Such re-excision surgery can result in poorer cosmetic results for breast reconstructions, and positive margins are associated with increased local and distant recurrence of the disease [8,9,10,11]. The economic impact is also significant. In addition to the costs associated with the re-excision, there is increased patient expense in clinical follow-up care. Institutions are also negatively impacted as their reimbursements are significantly less for repeat procedures (for many centers, a re-excision is a net financial loss), and valuable resources are tied up with unprofitable repeat surgeries.

Re-excision rates may depend on margin status and vary from 6.3 to 85.9% in the US [12]. What is not emphasized in most of these studies is that local recurrence, an indication of “surgically missed” cancerous tissues, still occurs in an average of 5–16% of patients with pathologically adjudged clean margins, indicating that one or more regions of tumor were not detected during pathological sampling [9,13,14]. Indeed, some studies indicate that, for such patients with pathologically assessed negative margins, local recurrence and secondary surgeries varied widely among surgeons (from 0% to 70%) and institutions (from 2–21%) [12]. This variation is likely because sampling only assesses a small portion of the margin of any given specimen. Recently, SSO-ASTRO consensus guidelines have highlighted the importance of obtaining negative margins, defined as non-invasive breast carcinoma or DCIS at ink, to optimize local control [4]. The consensus emphasizes that, although negative margins (no ink on tumor) minimize the risk of local recurrence, the routine practice of obtaining wider negative margin widths than no ink on tumor does not appear to further reduce local recurrence rates [4]. Together, these studies demonstrate the current unmet clinical need for technologies that rapidly and globally identify cancerous tissues in the margins of BCS specimens (i.e., at the surface of the lumpectomy samples) that can be used to guide surgical resections in real time.

In response to this demand for techniques to assay tumor margin infiltrates during resection, several technologies have been developed, yet few have become routinely used and each has significant weaknesses. Frozen section analysis is not accepted as part of the standard of care for assessing margins intra-operatively because of the difficulties of processing tissues with high fat content, such as the breast, as well as the added time, the increased cost, and the sampling rate limitations (personal communications with pathologists at University Hospitals Case Medical Center) and Refs. [7,15]. Touch prep cytology can rapidly assess the entire surface of the excised specimen but requires tumor cells to detach from the surface. Intraoperative radiography of the excised tissues, which has a sensitivity of 73% and a specificity of 49%, cannot identify microscopic processes and is limited when the tumor boundary is poorly defined [16,17]. Radiofrequency spectroscopy has a low sensitivity (71%) and specificity (68%), can only sample small areas at once (0.7-cm diameter), and requires comparison with pre-acquired libraries of known positive and negative signals [18]. Optical coherence tomography has high resolution, good sensitivity, and specificity but is subject to artifacts introduced by cauterization or blood vessels. It also requires training, and the existing technology cannot sample the entire tissue surface in a reasonable amount of time [19]. More recently, Dune Medical Inc. (Alpharetta, GA, USA) has developed a radio-impedance device for ex vivo detection of cancer in lumpectomy margins. This technique is only able to regionally sample the lumpectomy, and it only improves re-excision rates modestly [18]. Although intraoperative ultrasound-guided excision combined with intraoperative assessment of macroscopic pathologic and ultrasound margins in non-palpable invasive cancers has shown its practicality for conservative breast therapy [20,21], ultrasound typically cannot visualize in situ or multifocal cancers [22,23].

Thus, due to the limited and variable ability of these approaches to identify margin infiltrations, the difficulties associated with each technique or the added surgical time, these aids are seldom used to guide resections [14], although their development emphasizes the unmet clinical need for real-time assessment of BCS margins. 

Consequently, imaging tools are emerging to improve the quality of surgical resection: more specifically, technologies and approaches that enable surgeons to identify and assess the status of tumor margins with accuracy. In this regard, the use of targeted, fluorescent molecular probes to identify cancer tissue has shown promise for fluorescence image guided surgery (FIGS) [24,25,26,27]. Our own work has focused on the creation of quenched activity-based and substrate-based probes that have the potential to precisely delineate tumor margins and to detect residual, microscopic disease during the surgical procedure [28,29,30,31]. To achieve this objective, we have focused on targeting activated cysteine cathepsins. These proteases hydrolyze peptide bonds in proteins and are involved in various physiological processes, such as digestion, cell cycle regulation, proteolysis, extracellular matrix remodeling, apoptosis, and pro-protein activation. Activated proteases are upregulated in the cancer cells and, due to increased infiltration of immune cells, the cancer microenvironment of virtually all solid tumors. They are also differentially expressed in marginal tissue where tumor growth is most active [32,33,34,35,36,37,38,39,40,41]. Several studies have demonstrated the utility of quenched near-infrared (NIR) fluorescent molecular imaging probes for imaging tumor proteases in animal models of cancers [42,43,44,45].

More recently, we have focused on the development of an optimized, targeted contrast agent with enhanced contrast for multiple tumor types that is compatible with the existing approved imaging systems and can integrate easily into current surgical workflows. The industry standard fluorophore for these systems is indocyanine green (ICG). Our design is a cathepsin-targeted ICG-based contrast agent that exploits a latent lysosomotropic effect (LLE), inducing, upon cleavage, probe accumulation in lysosomal compartments. The result is significantly increased signal strength and duration over other approaches. We have demonstrated the suitability of these substrate-based probes (SPB) for clinical translation [31], as well as in tissue culture and in subcutaneously implanted tumors in mice [46]. In addition, fluorescent SBPs can be rendered optically silent by the addition of a quenching moiety that is lost upon reaction with the protease target of interest, ensuring that the probes only produce a signal in tumor tissue when acted upon by an enzyme activity associated with a tumor or its surrounding margins [47]. In our more recent work, we have developed a method for topical application of imaging probes to tissues using topical application of both quenched activity-based probes [28,29,48] and a third generation quenched SBP [31,49]. Topical application into the surgical cavity to identify remaining or “missed” cancer following lumpectomy has the potential to reduce positive surgical margins and integrates well into the surgical workflow. Ease of application and rapid activation of probe fluorescence (within minutes) make this approach a suitable tool for identification of tumor-associated proteases during BCS.

However, our research has shown that the central issue for topical application of imaging probes is gravity, which causes dripping and pooling of the liquid carrier (DMSO or saline) of the imaging probe at the bottom of the surgical cavity. Such accumulations can potentially lead to non-uniform distribution of the probe, resulting in false positives (pooled areas) and false negative signals (area without probe). To mitigate these issues, we propose the use of a carrier exhibiting a higher viscosity than DMSO or saline to effectively “immobilize” the probe in the surgical cavity for a sufficient period to ensure accurate probe activation congruent with diseased tissue.

In the current study, we tested different formulations of the Pluronic F127 nonionic surfactant polyol that belongs to the class of copolymers known as poloxamers. These copolymers become viscous at body temperature, resulting in uniform probe coverage of the tissue to which the probe has been applied. Here, we show that one of our gel formulations of Pluronic F-127/Kolliphor^®^ P407, a cGMP pharmaceutical-grade poloxamer, is a suitable carrier for the AKRO-QC-ICG imaging probe when used for topical application to demarcate normal and cancerous tissue in the surgical wound in vivo.

## 2. Materials and Methods

### 2.1. Performance Requirements

Performance requirements for activation time and signal persistence were generated from our discussions with surgeons and pathologists and were as follows: maximum signal must be achieved in 15 min or less with a signal-to-noise ratio of 2X or better over background; retention time (which we define as the time of “washout” from tissues when signal drops below 2X background) must exceed 30 min, with sensitivity and specificity >85%.

### 2.2. Gel Formulation and Studies

We tested Pluronic F-127 (BASF, CAS number: 9003-11-6) and pharmaceutical grade (Lutrol^®^ F-127, Poloxamer-407, Kolliphor^®^ P-407) gels with differing formulations. All gels were liquid at 4–8 °C. Since DMSO is essential for solubilization of the AKRO-QC-ICG, it has always been integral to the probe formulation. To maximize practicality of the gel formulation, our gel of choice, #30 (17% *v*/*v* gel and 20% *v*/*v* DMSO), was prepared as follows: 17.0-g of Pluronic F-127 or Kolliphor^®^ P-407 were dissolved in 70 mL of saline, autoclaved (liquid cycle), cooled, and then mixed with 20 mL of sterile DMSO (0.22 nm nylon filter). The final volume was adjusted to 100 mL with sterile saline. This mix was stored in a refrigerator before homogenization with a concentrated AKRO-QC-ICG DMSO stock (5 mM) solution.

### 2.3. Gel and Imaging Gel Application

Imaging gels with a lower concentration of Pluronic F-127 were topically applied onto both the tumor and normal tissue by airbrush (Iwata Eclipse Airbrush Kit, Portland, OR, USA). Imaging gels with a higher concentration of Pluronic F-127 were topically applied using cotton swabs.

### 2.4. Cell Culture Preparation for Orthotopic Implants

Human MDA-MD-468 and -231 triple-negative breast adenocarcinoma cells (ATCC) were grown in DMEM + 10%FBS, 1% penicillin/streptomycin, 10 μg/mL insulin at 37 °C, and 5% CO_2_. Immediately following cell harvesting, animals were anesthetized with isoflurane (Abbot) and inoculated. Breast adenocarcinoma cells (1 × 10^6^ in sterile PBS) were injected into the 4th mammary fat pad of mice in a 1:1 ratio with Matrigel (BD Bioscience).

### 2.5. Animal and Tumor Model

All animal procedures were performed according to Case Western Reserve University’s Institutional Animal Care and Use Committee (IACUC) approved protocols #2015-0033. Animals were fed a special rodent diet (Harlan Laboratories, Inc., Itingen, Switzerland) to reduce potential for auto-fluorescence. The study dataset consists of 12 nude mice with 12 orthotopic human breast carcinomas derived from MDA-MB-231 (n = 2) and MDA-MB-468 (n = 10) cell lines and 10 NSG patient-derived xenografts (PDX) mice implanted with TM000098 (n = 3), TM000091 (n = 2), TM000089 (n = 2), and TM000284 (n = 3) human patient breast adenocarcinomas to create PDX models. During surgery, abdominal skin was removed, followed by cutting off the visible tips of the tumor xenograft; minor bleeding was stopped by using a Bovie Cautery Kit. A substantial portion of both tumor xenografts was located under the abdominal wall and was not resected (PDX model).

### 2.6. Histology of Tissue Samples

Tissues were harvested and immediately frozen in an optimal cutting temperature compound (Tissue Plus, Scigen^®^, Paramount, CA, USA). Frozen blocks of tumor along with surrounding normal tissue were sectioned (10 μm) at −20 °C (Leica-CM3050S). The cryosections were then fixed and stained with hematoxylin–eosin (H&E) [28,29].

### 2.7. Imaging of Animal BCa Models

All bright field, fluorescent, and merged (bright field and fluorescent) images were taken by a Curadel Lab-FLARE^®^ RP1 imaging system with 800 nm filter set (Curadel, LLC, Natick, MA, USA) or Spectrum IVIS^®^ camera with an ICG filter set (PerkinElmer, Inc., Waltham, MA, USA) and analyzed using Resvet Imaging software, v.4.0 (Curadel) or Living Image^®^ Analysis software (Perkin Elmer, Waltham, MA, USA). Fluorescent images were viewed with a Leica-DM4000B microscope (Leica Microsystems, Buffalo Grove, NY, USA) and analyzed with QCapturePro-7 software, v.5.0 (QImaging, Surrey, BC, Canada).

### 2.8. Imaging of the Human BCa Tissue

All bright field, fluorescent, and merged (bright field and fluorescent) human tissue images and studies were conducted in accordance with the Declaration of Helsinki. Written informed consent was obtained before any study-related procedure was performed, and the research protocol was approved by ethics committee (code#058, approved date—7 April 2021) of the Haaglanden Medical Center (HMC), the Netherlands. To investigate the ability of the probe to visualize human BCa tissue, AKRO-QC-ICG imaging gel#30 was applied onto the freshly resected tissue specimen of one BCa patient who underwent lumpectomy at the HMC (The Hague, The Netherlands). Patient and tumor characteristics are summarized in Supplemental Table S1. Briefly, directly after resection, the specimen was brought to the HMC Pathology Department. According to HMC hospital protocol, the specimen was inked, cooled at −80 °C for 5 min, and sliced into 3-mm-thick slices. AKRO-QC-ICG imaging gel (1 mL, 50 μM final) was applied in one layer using a cotton swab onto the tissue slice with the macroscopically largest tumor diameter. After 10 min of incubation, the imaging gel was triple washed with sterile saline and the tissue slice was dried with sterile gauze. Subsequently, fluorescence images were obtained using the Pearl Trilogy Imaging System (LI-COR, Lincoln, NE, USA). The complete tissue slice was then fixed with formalin, quartered, and embedded in paraffin. One 4 μm slide per formalin-fixed, paraffin-embedded tissue block was stained for routine H&E histology and digitized by scanning using the Philips IntelliSite Pathology Solution (Philips Electronics, Eindhoven, The Netherlands). Tumor-positive areas were delineated by a board-certified pathologist (Hans Marten Hazelbag) blinded for the fluorescent signal.

### 2.9. Statistics (Mouse Studies)

Sensitivity and specificity were estimated as binomial proportions and exact 95% confidence intervals were calculated as before [30]. The PDX model dataset consisted of 10 tumor samples. Within a tissue cut, one or more normal (non-cancer) and tumor (cancer) areas were defined, and, for each area (spot), the presence of black ink, indicating fluorescence, was recorded. From the 10 samples, a total of 10 histology slides (cuts) were made, and a total of 36 areas (spots) were selected. Of these 36 areas, 16 were marked as having tumor, and 20 marked as having no tumor. In per spot analysis, these spots are treated as independent observations. The nude mice dataset consisted of 12 tumor samples. Within a tissue cut, normal (non-cancer) and tumor (cancer) areas were defined, and, for each area (spot), the presence of black ink, indicating fluorescence, was recorded. From the 12 samples, a total of 12 histology slides (cuts) were made, and a total of 24 areas (spots) were selected. Of these 24 areas, 12 were marked as having tumor, and 12 marked as having no tumor. In per spot analysis, these spots are treated as independent observations. The analysis of sensitivity uses only those spots classified as having tumor, and the analysis of specificity includes only those spots classified as having no tumor. Sensitivity and specificity are estimated as binomial proportions, with exact 95% confidence interval also calculated. All numerical results are expressed as mean ± SD. Descriptive statistics and significant differences between groups were analyzed by using two-tailed Student’s *t* tests. A *p* value < 0.05 was considered statistically significant for all comparisons.

## 3. Results

### 3.1. AKRO-QC-ICG Specifically Accumulates and Is Activated in Human BCa Cells

**Figure 1:**
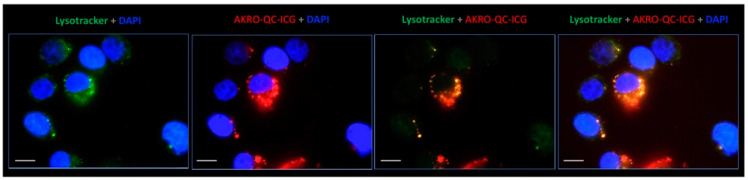
AKRO-QC-ICG accumulates in lysosomes and becomes fluorescent in the human BCa cells in vitro. Representative live cell fluorescence microscopy images of the human breast MDA-MB-486 cancer cell monolayer incubated for 30 min with 1 μM of quenched substrates followed by washout. ICG fluorescence of probe is false-colored red, lysotracker (lysosome selective stain) is false colored green, blue is DAPI (nuclear stain), and yellow/orange corresponds to lysosome/ICG co-localization. Magnification—100× with immersion (Leica-DM4000B). Scale bar—10 μm.

To validate the LLE for breast cancer cells, we measured probe activation in MDA-MB-468 human breast cancer cells in vitro. We observed strong probe fluorescence in lysosomal compartments of cells incubated with the quenched substrate, suggesting that this probe is indeed activated by lysosomal cysteine proteases in this human breast cancer cell line (Figure 1). Labeling in the live cells was selective to lysosomes, as confirmed by the strong colocalization of the probe with the lysosome-specific marker Lysotracker Green. A similar labeling pattern of AKRO-QC probes was observed earlier for a macrophage-derived cell line RAW264.7 [46]. The presence and fluorescence of AKRO-QC-ICG probe in lysosomes of live cells from different origins suggests that the probe is internalized, accumulated, and becomes fluorescent only in the presence of activated target enzymes.

### 3.2. Human Breast Cancer Cell Lysate Initiates Specific Fluorescence of the AKRO-QC-ICG Probe In Vitro

**Figure 2:**
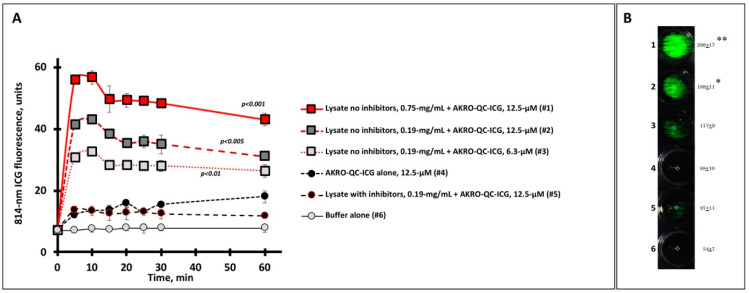
Human BCa cell lysate initiates specific fluorescence imaging of AKRO-QC-ICG probe in vitro. (**A**)—Time course of ICG fluorescence imaging of cancer-cell-associated cysteine cathepsins (lysate) mixed with substrate (AKRO-QC-ICG). Tecan fluorescent scanner was used to assess ICG fluorescence in 4 wells per sample for different mixes, probe alone, or buffer alone (100 μL per well, 96-well black flat bottom plate) in three experiments. Irreversible cysteine protease inhibitor K777 was used as a control for specificity of fluorescence. Vertical bars—SD. (**B**)—Representative sample of ICG FL in AKRO-QC-ICG/MDA-MB-468 cancer cell lysate mixes after 10 min incubation. Data presented as mean fluorescence ± SD in 3 wells per sample. Number of wells corresponds to the number of samples tested in (**A**). Quantification of well signal in arbitrary units after 10 min of incubation is presented to the right of each well. Imaging of lysate spots was performed using the Curadel Lab-Flare RP1 with 800 nm filter set and Curadel Resvet Imaging software. Notes: (1) the levels of fluorescence of all lysates without inhibitor were significantly higher as compared to controls (wells #4–#6) in (A); *—*p* < 0.05, **—*p* < 0.01 compared to controls (wells #4–#6) in (B) by Student’s t-test in three independent experiments both for (A) and (B)).

To test the specificity of AKRO-QC-ICG for proteases from cancer cells, we assessed AKRO-QC-ICG activation after incubation with BCa cell lysates in vitro. We observed rapid probe activation, as measured by accumulation of ICG fluorescence signal, within 10 min, which was dependent on the concentration of lysate used (Figure 2). The intensity of the fluorescent signal reached a maximum 10 min after incubation and was sustained for 60 min. The levels of ICG fluorescence resulting from the highest extract concentration were seven-fold higher as compared to controls (probe alone (#4), probe with protease inhibitor (#5), or buffer alone (#6)).

### 3.3. Thermo-Sensitive Gel Formulation of AKRO-QC-ICG Enables Uniform Topical Distribution of Probe along the Surfaces of the Surgical Cavity

Topical application of an imaging probe onto all surfaces of a surgical cavity is potentially challenging. A surgical cavity has a complex three-dimensional profile. Consequently, topical application of any imaging probe that is dissolved in a liquid carrier and applied onto the sloping irregular sides of a surgical wound results in pooling and dripping. Probe flow and pooling effects lead to false negative zones at the top of the wound (where no probe remains) and false positive areas in the wound bed (where there is excess probe accumulation and, consequently, high autofluorescence). To mitigate this issue, we developed a thermo-sensitive pluronic-based gel that could be applied in a liquid- to semi-solid state that was then thermally activated to congeal when it meets body tissue. More than 40 different formulations of thermosensitive soluble-gel Pluronic F-127 (as a carrier), DMSO (as a solvent and carrier), and AKRO-QC-ICG (as an imaging probe) were tested using an inclined plane heated to 37 °C. (Supplemental Figure S1). Some formulations dried too quickly, while others had insufficient viscosity and pooled at the bottom of the surface of a slope. 

**Figure 3:**
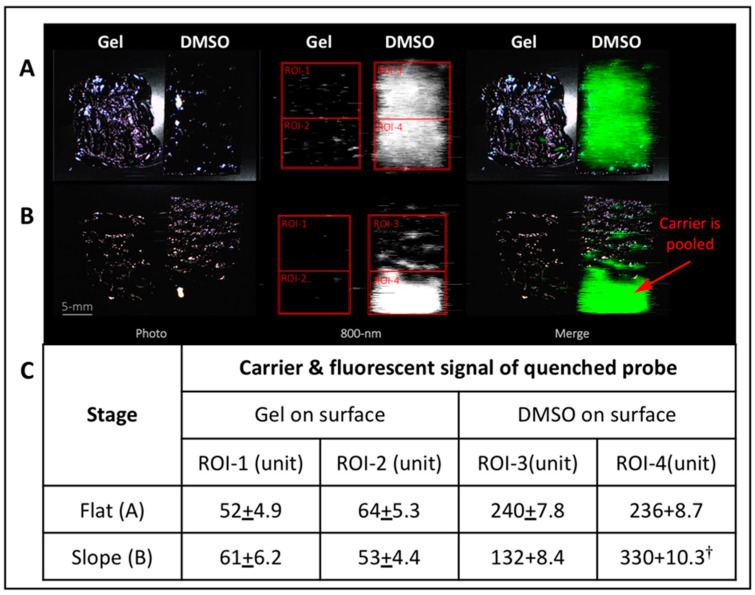
Thermo-sensitive Pluronic F-127 hydro-gel#30 possesses better carrier characteristics for “no drip” topical applications of AKRO-QC-ICG imaging probe under force of gravity. Analysis of behavior of carriers of the AKRO-QC-ICG imaging probe. Both AKRO-QC-ICG (final 5 μM) dissolved in 50 μL of Pluronic F-127 hydro-gel#30 and a paper applicator impregnated with AKRO-QC-ICG (final 5 μM in 50 μL of DMSO) were topically applied to a plastic surface that was pre-heated at 37 °C. (**A**)—Distribution of the AKRO-QC-ICG in the carriers on the flat surface. (**B**)—Distribution of the AKRO-QC-ICG in the carriers on the surface with a 30 degree slope after 1 min. Scale bar 5 mm. (**C**)—Analysis of the levels of auto-fluorescence of AKRO-QC-ICG at the upper and bottom quadrant of the applied carrier in the regions of interest (ROIs). Data were normalized to background. Notes: †—*p* < 0.05 compared to ROI-3 in (**B**) with slope (Student’s *t*-test in three independent experiments). Imaging and analysis—Lab-FLARE^®^ imaging system with 800 nm filter set and Resvet Imaging software (both Curadel, LLC, Natick, MA, USA). It should be noted that the lower concentration of probe used here is quenched by gel formulation, but not when formulated in 100% DMSO. Quantitation of signal reveals lack of probe movement when formulated using gel#30.

Our results found that a mixture of 17% *v*/*v* Pluronic F-127 and 20% *v*/*v* DMSO in sterile saline (gel#30) possessed the best characteristics for “no drip” topical applications of AKRO-QC-ICG (Supplemental Figure S2). To test this gel formulation for application of AKRO-QC-ICG, both gel#30 containing AKRO-QC-ICG and a paper applicator with equal amounts of AKRO-QC-ICG in DMSO were applied onto the heated surface sloped at 30 degrees. Figure 3 shows that probe in gel#30 remained moist and in place, while AKRO-QC-ICG applied in 100% DMSO flowed down the inclined slope, accumulating at the bottom of the paper applicator. While both gel#30 and DMSO as carriers showed equal distribution of the 800 nm fluorescence when the surface for topical application was flat (Figure 3A), rapid accumulation of fluorescence was found at the bottom of the sloped surface for DMSO-only formulation (Figure 3B). When AKRO-QC-ICG was formulated with DMSO and gel#30, gravity did not influence the gel distribution. This finding was confirmed as we measured equal and uniform fluorescence at the upper and lower portions of the applied gel and DMSO on paper (ROIs 1–4, Figure 3B). These data also demonstrated that the AKRO-QC-ICG was further quenched by the gel formulation compared to the DMSO formulation.

#### 3.3.1. The Gel Formulation of AKRO-QC-ICG Enables the Visualization and Discrimination of Human BCa from Normal Mouse Tissue In Vivo

The gel formulation of AKRO-QC-ICG was tested in vivo using two animal models: (1) orthotopic human MDA-MB-231 and -468 breast cancer cell xenografts in nude mice; and (2) human breast cancer cell orthotopic-patient-derived xenograft (PDX) in NSG mice. In the nude mouse model, MDA-MB-231 and -468 breast cancer cell were inoculated into the mammary fat pad of mice to generate tumors. After the tumors reached the appropriate size of 100 mm^3^, they were surgically exposed, and gel formulated AKRO-QC-ICG was topically applied to the entire wound area encompassing both normal and tumor tissues. At various times, the tissue was washed free of probe, and noninvasive imaging was performed. 

**Figure 4:**
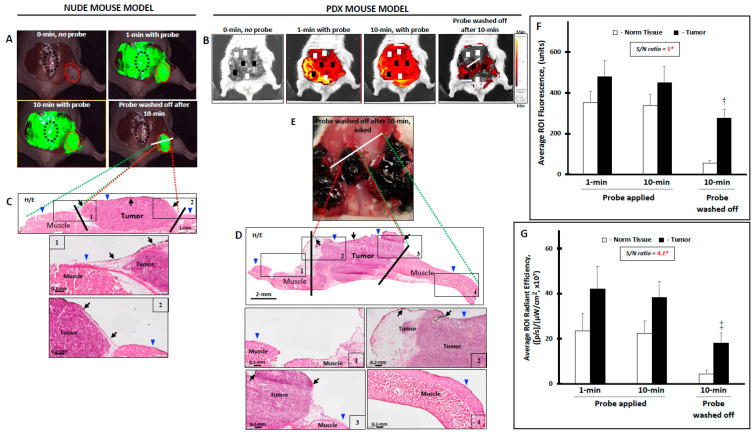
Topical application of AKRO-QC-ICG imaging Pluronic F-127 hydro-gel#30 visualizes and helps to demarcate the human BCa tissue from normal mouse tissue in vivo. (**A**)—The levels of ICG fluorescence in the surgical cavity of athymic nude mouse with MDA-MB-468 human breast cancer cell xenograft. Tip of the tumor xenograft (up to 40% of visible tumor) was cut off, bleeding was cauterized, and AKRO-QC-ICG imaging hydro-gel#30 (AKRO-QC-ICG = 50 μM final) was topically applied onto all surfaces of the abdominal muscle wall along with tumor xenografts. After 10 min of incubation, the imaging gel was washed away with sterile saline from everywhere, including tumor, and surgical cavity was imaged. Based on this 10 min image, only high fluorescent zones were inked by a black pathological ink. White line—plane of section. (**B**)—The levels of ICG fluorescence in the surgical cavity of NSG PDX mouse with xenografts that were grown from the human breast cancer cells isolated from patient TM00098. Tips of the tumor xenografts (up to 40% of visible tumor) were cut off, bleeding was cauterized, and AKRO-QC-ICG imaging hydro-gel#30 (AKRO-QC-ICG = 50 μM final) was topically applied onto all surfaces of the abdominal muscle wall along with tumor xenografts. After 10 min of incubation, the imaging gel was washed away with sterile saline from everywhere, including tumor, and surgical cavity was imaged. Based on this 10 min image, only high fluorescent zones were inked by a black pathological ink. White line—plane of section. (**C**)—Bread-loaf section and H&E histology of (**A**); blue triangles indicate the surface where the imaging gel was applied. Inserts-1 and -2 show borders between normal tissue and cancer (bottom—with high magnification). Black arrows—ink in the tumor zone. Black lines demarcate the cancer zone border. (**D**)—Pathology ink on the tumor surfaces of the surgical cavity that was used to mark the areas of high ICG-fluorescence in (**B**). (**E**)—Bread-loaf section and H&E histology of (**B**), blue triangles indicate the surface where the probe was applied. Inserts-1 and -4 (upper panel) show normal tissue and inserts-2 and -3 show borders between normal tissue and cancer (bottom panel—with high magnification). Black arrows—ink in the tumor zone. Black lines demarcate the cancer zone border. (**F**)—The average levels of fluorescence in the ROIs of the tumor (red dotted circles) and normal (black dotted circles) tissue as represented in the surgical cavity in (**A**) for all athymic nude mice, n = 12. (**G**)—The average levels of fluorescence in the ROIs of the tumor (black squares) and normal (white squares) tissue as represented in the surgical cavity in (**B**) for all NSG PDX mice, n = 10. Notes: (1) *—normalized to 0 min pre-image = background; (2) †—*p* < 0.001 to normal tissue at 10 min (n = 12), when the imaging gel was washed off in (**F**); ‡—*p* < 0.005 to normal tissue at 10 min (n = 10), when the imaging gel was washed off in (**G**) by Student’s *t*-test; 3) Vertical bars—SD; 4) cameras: Curadel Lab-Flare RP1 and Curadel Resvet software in athymic nude mice and IVIS^®^ Spectrum in vivo imaging system and software in NSG PDX mice.

When AKRO-QC-ICG gel#30 was applied to the surgical cavity (tumor and normal surrounding tissues), there was uniform fluorescence resulting from the high probe concentration and autofluorescence. At stipulated times, the probe was removed by saline wash and imaged. Imaging demonstrated that the portion of the probe that was applied over tumor tissue was rapidly activated and produced fluorescence that remained after saline washes. In contrast, after washing, no fluorescent signal was measured for probe that was applied to non-cancerous tissues. Sufficient contrast to enable discrimination of tumor from the surrounding normal tissues was achieved within 10 min of probe application (Figure 4A). The fluorescent signal from the tumor was significantly higher as compared with normal surrounding tissue after rinsing, yielding a signal-to-noise (S/N) ratio equal to 5.0 (Figure 4F).

In the PDX NSG mouse model, a few isolated xenografts sometimes developed penetration into the abdominal cavity. Topical application of gel#30-formulated AKRO-QC-ICG onto normal and tumor tissue also demonstrated accumulation of the cathepsin-induced probe-dependent fluorescent signal at the areas of tumor rather than at the surrounding normal tissue (Figure 4B) when the surgical area was washed after 10 min incubation with probe. As with the nude mice, we found that the average fluorescent signal in tumor tissue was significantly higher than that in normal tissue. The S/N ratio was equal to 4.1 (Figure 4G) for this model; the lower fluorescent yield could be related to massive tumor invasion into the abdominal cavity and the small separation between tumor xenografts. In general, we found that this gel formulation was a good solvent/carrier for AKRO-QC-ICG with a desirable thermo-reversible sol–gel transition and appropriate mechanical strength necessary for even distribution of AKRO-QC-ICG on the surface of tissue.

Following initial imaging, the fluorescent areas were painted with pathology ink and the tissues underwent histology. The slides were read by a pathologist and the association of cancer with fluorescence (i.e., pathology ink) was assessed, as in Figure 4E. Analyses of the data to estimate sensitivity and specificity were conducted both on a per-spot basis and on a per-animal basis for both the nude and NSG mice used to generate Figure 4. Analysis of these data revealed positive and negative predictive values of tumor detection after topical application of the imaging gels in the nude mice as 1.00 (95% CI = 0.699–1.000) and 0.917 (95% CI = 0.598–0.996), respectively. Table 1 shows the levels of sensitivity and specificity of our method, derived from the 12 nude mice.

Similar results were obtained for per-animal analysis. For the per-animal analysis, results from multiple slides and areas (spots) from the same sample were combined into a single record. Positive and negative predictive values of tumor detection after topical application of the imaging gels in the PDX mice were analyzed separately and were found to be 1.00 (95% CI = 0.759–1.000) and 0.950 (95% CI = 0.730–0.997), respectively. Table 2 shows the levels of sensitivity and specificity of our method, derived from the 10 PDX mice. 

#### 3.3.2. The Behavior of the AKRO-QC-ICG Gel Formulation on the Surface of the Human BCa Tissue In Vivo

**Figure 5:**
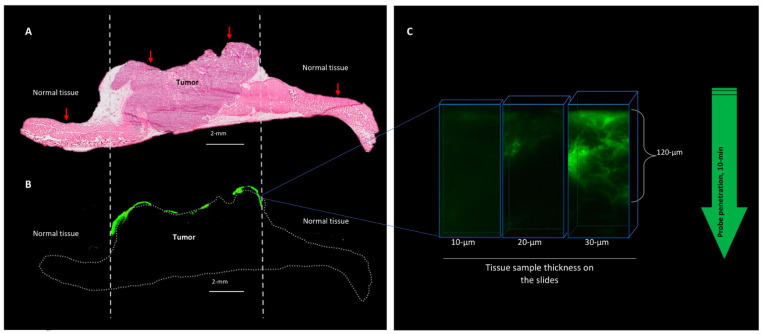
After topical application, AKRO-QC-ICG probe penetrates tissue to the depth of a few cancer cells. (**A**)—H/E histology of the PDX cancer xenograft with surrounding normal tissue (abdominal wall muscles). Vertical dotted lines indicate tumor borders. (**B**)—Fluorescent scan of consecutive tissue slide represents AKRO-QC-ICG-induced fluorescence (false green color), indicating that the probe was activated only at the area of the fresh cut surface of the tumor without a tendency to spread to surrounding tissues. Dotted line—contour of the sample. (**C**)—Fluorescent microscopy consecutive tissue sections of different thickness indicate that AKRO-QC-ICG penetrates from the surface (where it was applied) to a depth of up to 120 μm in 10 min. Thicker tissue sections were required to visualize probe fluorescence in tissue, avoiding the need for inking fluorescence prior to tissue preparation. For these studies, mice were treated with AKRO-QC-ICG imaging gel#30 and imaged as in Figure 3. Next, tissue was snap frozen and consecutive slides were sliced for routine histology, fluorescent scan (Odyssey CLx, resolution = 24 μm, 800 nm filter set, Li-Cor) in (**B**) and microscopy (Leica, NIR filter set) in (**C**). Vertical red arrows indicate the surface of application and direction of future penetration of the probe in (**A**).

To determine the penetration and diffusion of the AKRO-QC-ICG gel formulation when delivered topically, tumor xenografts and surrounding normal tissue were washed of probe after application and incubation. They were then imaged, dissected, and frozen sections were prepared. Consecutive sections underwent fluorescence imaging and H&E staining. H&E staining (Figure 5A) and fluorescent scanning of the frozen slides with maximum resolution (Figure 5B) showed that the fluorescent signal was localized on the surface of the tumor tissue. Fluorescent microscopy of the tumor tissue determined that gel#30-formulated AKRO-QC-ICG penetrated the tumor tissue and was activated at the depth of a few cancer cells (120 μm) 10 min after topical application (Figure 5C).

We also assessed the behavior of the imaging gel on the surface of the freshly excised mouse organs (Supplemental Figure S3) and found that the inner surface of the spleen, liver, kidney, and lung capsule were fluorescent-free after topical application of the gel#30-formulated AKRO-QC-ICG probe as compared to the human breast cancer xenograft. These data suggest the formulation is selective for targeting cancer tissue.

#### 3.3.3. Gel Formulated AKRO-QC-ICG can Detect BCa in an Excised Human Lumpectomy Sample

**Figure 6:**
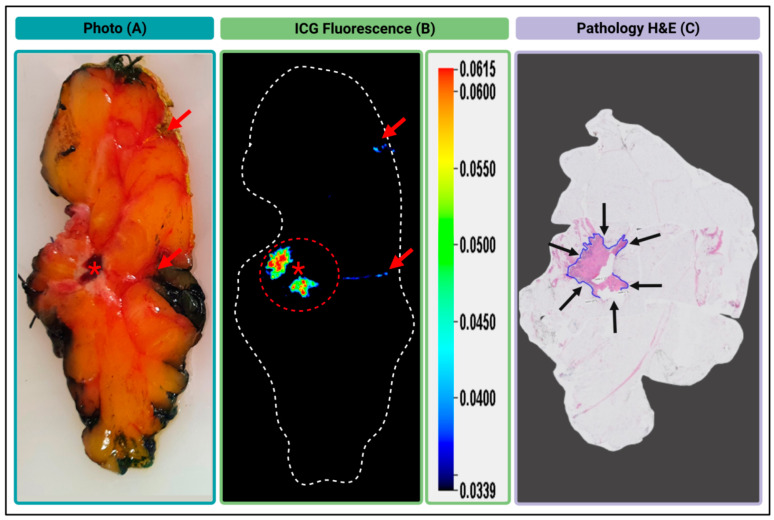
Ex vivo fluorescent imaging of the human BCa lumpectomy tissue sample. (**A**)—Color photo of the surface of the fresh cut of the human lumpectomy sample. Red asterisk indicates a blood clot. (**B**)—Image of 6QC-induced cathepsin-dependent ICG-fluorescence of the sample surface after topical application of AKRO-QC-ICG imaging gel (800 nm). The red dotted circle shows a high fluorescent ICG signal that corresponds to the tumor region of the sample. (**C**)—H&E histology of the sample at low magnification. Blue line and black arrows represent the tumor area. Notes: red arrows indicate spots of the yellow pathological ink in (**A**) and non-specific fluorescence in (**B**) on the surface and edge of the sample. Imaging—Pearl Trilogy Imaging System.

The overall goal of these studies is to develop a probe technology that can be topically applied to the surgical cavity after lumpectomy and, in near real time, identify any remaining BCa in the surgical cavity. This approach should allow for its immediate removal with consequent reduction in secondary surgeries. To achieve and validate a real-time intraoperative capability of gel#30-formulated AKRO-QC-ICG for detecting BCa in human breast, gel#30-formulated probe was applied topically onto a fresh cut of a human lumpectomy sample ex vivo in Figure 6. The white light (Figure 6A), ICG-fluorescence (Figure 6B), and pathology (Figure 6C) images of the 3-mm-thick human breast lumpectomy cancer tissue slice are shown. The tumor area in Figure 6C corresponded to a high fluorescent ICG signal in Figure 6B. Only the part of the tumor that was covered by a blood clot (as shown in Figure 6A) did not show probe activation (Figure 6B). Further analysis of ICG fluorescence from the sample revealed that an S/N ratio was relatively high and non-specific fluorescence was found at the edge of the sample where yellow pathology ink was used (Figure 6A,B). It is important to note that, in contrast to green or black color pathology inks, the yellow ink was found to display auto-fluorescence at 800 nm, and, hence, caution is advised regarding its use in fluorescent imaging of the pathology sample ex vivo (Supplemental Figure S4).

**Figure 7:**
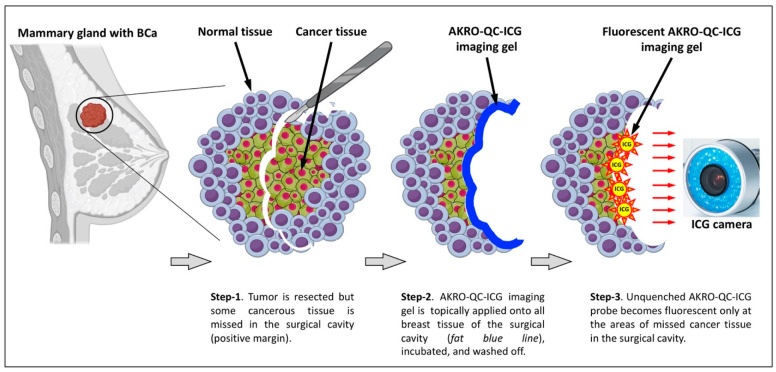
The scheme of the approach proposed for topical application of AKRO-QC-ICG imaging gel into the surgical cavity to detect missed cancerous tissue.

Here, we use the cartoon (Figure 7) to better illustrate our approach for topical application of AKRO-QC-ICG imaging gel for the detection of BCa remnants in the surgical cavity. The color photo in Figure 6A represents a side/bottom of the surgical cavity with a BCa tumor remnant, as shown in Figure 7 Step 1.

Next, Figure 4A,B (both 1 and 10 min with probe) represent Step 2 in Figure 7, when the AKRO-QC-ICG imaging gel is distributed onto the surface of the surgical cavity to make physical contact to the BCa hidden remnants, if any. Figure 7 Step 3 corresponds to Figure 4A,B (probe washed off after 10 min) and Figure 6B, when the imaging gel is washed off, followed by detection of human-BCa-induced and AK-RO-QC-ICG-gel-related ICG fluorescence, if any, in the surgical cavity using the NIF camera. This capability may enable “real-time” surgical adjustments (such as additional tissue shaves) during a single surgical procedure in the operating room. 

## 4. Discussion

We demonstrated that, after topical in vivo application of a fluorescent molecular probe to the surgical cavity, BCa tissue can be imaged and residual disease reliably detected. Meeting clinically derived goals for this project required activation to full signal within 15 min of application, persistence of such signal for at least 30 min, and sensitivity and specificity greater than 85%. Formulation of the probe in a poloxamer gel enabled us to achieve activation time within the clinically mandated 15 min time frame with high sensitivity and specificity and peak signal persisting for 30 min (data not shown). While this project did not attempt to determine a limit for image resolution, our earlier studies have indicated that single-cell resolution is possible.

As stated above, the probe is activated by cathepsin proteases that, in the context of the tumor microenvironment, are predominantly derived from tumor-associated macrophages, as well as cancer cells. Because inflammation is a general marker for the presence of all solid tumors, general probes for inflammation, such as our cathepsin-activatable probe, are useful in the definition of these tumor margins. Moreover, even in cases where there is high background inflammation, our probe can still define tumor margins due to the high concentration of protease activity at the border between the cancerous and healthy tissue [28].

While, in vivo, topical use of this AKRO-QC-ICG has the potential to make a significant contribution to increasing the quality of BCS resections, there are challenges. Most notably, the probe takes several minutes to activate and achieve full signal strength. The probe must remain in contact with the tissue being interrogated not only during the application period but for a clinically relevant time frame to allow complete assessment of marginal status. However, processes such as dripping, pooling, or bleeding at the excision site could redistribute the probe both before and after activation. Our approach, therefore, was to explore ways to effectively “immobilize” the probe in the surgical cavity for a sufficient period to ensure activation. Our work, described here, indicates that poloxamer gels offer a potential solution. Poloxamers are synthetic nonionic triblock copolymers that have a central hydrophobic chain of polyoxypropylene, flanked by two hydrophilic chains of polyoxyethylene. These polymers exhibit thermo-reversible behavior in aqueous solutions and are widely used in pharmaceuticals [50]. By adjusting parameters such as composition, molecular weight, and concentration, gelation can occur at physiological temperature and pH [51]. Pharmaceutical-grade poloxamers are available in the USA under the brand name Pluronic^®^ (BASF). Pluronics are available in a wide range of forms, molecular weights, and compositions and are widely used in drug and gene delivery [52]. While hydrogels typically possess low viscosity and poor mechanical strength, which can make them less than ideal for the proposed application [53,54], testing of more than 40 gel formulations enabled us to select one that had characteristics (namely high viscosity and mechanical strength) that appeared to match well with our requirements. 

The potential of fluorescent molecular probes to improve surgical resection of a significant subset of solid tumors is well-documented, with several such probes currently entering, participating in, or completing formal clinical trials. Currently, all such probes require the systemic administration of pharmacologically significant dosages hours or days before surgery, with the attendant complication of additional laboratory tests and the risk of allergic reactions. Furthermore, such systemic probes may fail to reach non-vascularized tumors in marginal tissue around the main tumor, leading to incomplete surgical resection. The performance of probes targeted to specific tumor pathways/proteins may also be compromised by tumor heterogeneity. Such risks are mitigated for cathepsin-targeted probes, such as AKRO-QC-ICG, which are also activated by tumor-associated immune cells, i.e., macrophages [55].

AKRO-QC-ICG is a highly sensitive probe with promising potential for applications in optical surgical navigation [55] and has enabled detection of small flat mouse BCa cancer lesions in vivo after systemic delivery to the animals [55]. The ointment-like consistency of the gel-formulated AKRO-QC-ICG probe eliminates both the “dripping effect” of the imaging probe along the walls and “pooling effect” of the probe in the grooves and folds of the surgical cavity. We also found our preferred formulation of the imaging gel (gel#30) to be both easily applied and washable by saline, resulting in a cancer-specific fluorescent signal with a clinically acceptable signal-to-noise ratio in the surgical wound. Testing of gel#30 using a human lumpectomy sample showed a strong, robust, and specific ICG signal at the area of BCa (Figure 6). This single application of AKRO-QC-ICG imaging gel in its current formulation fully corroborates our previous clinical studies [30,31,49] and builds a strong case to support the clinical merit of topical application of imaging probes as an approach to optically visualize cancer tissue both ex vivo and in vivo. Most recently, de Jongh et al. [56] reported on the discrepancy between true negative margins and those obtained after using IV-administered FIGS. They concluded that back table observation of excised tissues during the surgery by pathology may be necessary to ensure clear surgical margins. Our approach directly reports on the presence of any cancer remaining within the surgical cavity and solves the issue of questionable margins.

An approach for topical application of AKRO-QC-ICG [28,29,30,31,49] for the detection of BCa tissue that is surrounded by normal tissue is illustrated in Figure 7. This scenario implies a presence of missed cancer tissue or avascular cancer cell clumps in the surgical wound after a standard of care or FIGS resection of primary breast adenocarcinoma (positive margins). The gel-formulated probe is then applied to the surfaces of the surgical cavity, incubated, and then removed with saline washes (Figure 7, Step 2). Finally, using a hand-held imaging camera (many are FDA-approved), the cavity can be inspected for a fluorescent signal and the cancerous tissue removed (Figure 7, Step 3). Re-application can be utilized to make sure that all the cancer was removed. Our results here suggest that the in vivo application of molecular probes may offer a solution to the problem of assessing marginal tissue after tumor resection [57,58,59,60], and we have identified and presented potential solutions to the challenges of using these probes in a clinical context. While topically applied probes can be used as a ‘stand-alone’ solution for probe-guided surgery, they can also be used in conjunction with systemically administered imaging probes in order to identify cancerous lesions, remove them, and check them for remaining cancer tissues.

This preclinical study suggests that utilization of thermosensitive soluble-gel Pluronic F-127, DMSO, and AKRO-QC-ICG mix topically to detect BCa in vivo in the surgical cavity has the potential to reduce re-excisions, as well as the false negative rates from pathological under-sampling, with consequent savings in healthcare costs and enhancement in patient quality of life.

## 5. Conclusions

Here, we demonstrate for the first time the utility of topically applied cathepsin-substrate-based fluorescent probe AKRO-QC-ICG in human BCa models. This probe is part of a thermosensitive imaging hydrogel mix that was formulated and tested specifically for topical application. Our results suggest that it reliably generates contiguous fluorescent images in vivo, allowing rapid and easily interpretable intraoperative surgical margin examination. Clinical adoption of the AKRO-QC-ICG technology may add advantages for surgeons to assess a surgical cavity in real time in the operating room.

## Figures and Tables

**Table 1:** AKRO-QC-ICG imaging Pluronic F-127 hydro-gel#30 detects the human breast cancer tissue in vivo with a high sensitivity and specificity in nude orthotopic mouse models.

Measure	Group Examined	n	Estimate	95% CI 2-Sided
Sensitivity	All samples	12	0.923 (12/12)	(0.621–0.996)
Specificity	All samples	12	1.000 (11/12)	(0.679–1.000)

Note: to assess efficacy of AKRO-QC-ICG hydro-gel#30 formulation for detecting cancerous tissue in vivo, the correlation between the inked areas, i.e., fluorescence, and pathologically confirmed cancer was determined as in Figure 4C. The dataset consists of 12 nude mice with orthotopic human breast carcinoma of MDAMB-231 (n = 2) and MDAMB-468 (n = 10) cell lines. Twelve athymic nude mice contributed 12 samples. Within a tissue cut, one normal (non-cancer) and tumor (cancer) area was defined. For each area (spot), the presence of black ink, indicating fluorescence, was recorded. From the 12 samples, a total of 12 histology slides (cuts) were made, and, on these 12 slides, there were a total of 24 areas (spots), 12 marked as having tumor and 12 marked as having no tumor. Analyses of the data to estimate sensitivity and specificity were both carried out on a per-spot basis.

**Table 2:** AKRO-QC-ICG imaging Pluronic F-127 hydro-gel#30 detects the human breast cancer tissue in vivo with a high sensitivity and specificity in NSG PDX mouse models.

Measure	Group Examined	n	Estimate	95% CI 2-Sided
Sensitivity	All samples	16	0.941 (16/16)	(0.692–0.997)
Specificity	All samples	20	1.000 (19/20)	(0.790–1.000)

Note: to assess efficacy of AKRO-QC-ICG hydro-gel#30 formulation for detecting cancerous tissue in vivo, the correlation between the inked areas, i.e., fluorescence, and pathologically confirmed cancer was determined as in Figure 4D. This PDX model dataset consists of 10 NSG mice with TM000098 (n = 3), TM000091 (n = 2), TM000089 (n = 2), and TM000284 (n = 3) human patient breast adenocarcinomas isolated cell xenografts as a PDX model. Since a mouse could have more than one tumor xenograft, the total number of samples was more than the number of animals tested. Ten NSG mice contributed 12 samples (tumors). Within a tissue cut, both tumor and surrounding normal tissues were captured. For each area (spot), the presence of black ink, indicating fluorescence, was recorded. From the 12 samples, a total of 12 histology slides (cuts) were made, and, on these 12 slides, there were a total of 36 areas (spots), 16 marked as having tumor and 20 marked as having no tumor. Analyses of the data to estimate sensitivity and specificity were carried out on a per-spot basis using pathological correlation of cancer with black ink, i.e., fluorescence.

## Data Availability

The data presented in this study are available in this article and Supplementary Material.

## Supplementary Materials

The following supporting information can be downloaded at: https://www.mdpi.com/article/10.3390/cancers14143459/s1, Supplemental Figure S1: Surface behavior of Pluronic F-127 hydrogel/DMSO mix depends on concentration of the Pluronic F-127 (formulations with lower concentrations of the hydrogel). Supplemental Figure S2. Surface behavior of Pluronic F-127 hydrogel/DMSO mix depends on concentration of the Pluronic F-127 (formulations with higher concentrations of the hydrogel). Supplemental Figure S3. AKRO-QC-ICG imaging gel becomes fluorescent (unquenched) only on fresh cuts of the human breast MDA-MB-468 tumor xenograft but not the normal organ tissues after topical application ex vivo. Supplemental Figure S4. Imaging of yellow color pathological ink auto-fluorescence with 800-nm emission filter set. Supplemental Table S1. Clinical and Pathological Characteristics of the Lumpectomy Sample.
