# Supplementary material for: Formulation of a Thermosensitive Imaging Hydrogel for Topical Application and Rapid Visualization of Tumor Margins in the Surgical Cavity
Source: Cancers (Basel). 2022 Jul 16;14(14):3459. doi: 10.3390/cancers14143459 (PMC9323389; doi:10.3390/cancers14143459)
Supplement: Supplementary file 1 [file cancers-14-03459-s001.zip › cancers-1796734-supplementary.pdf]

cancers

MDPI

Article

Formulation of a Thermosensitive Imaging Hydrogel for Topical Application and Rapid Visualization of Tumor Margins in the Surgical Cavity

Ethan Walker, Daan G. J. Linders, Eric Abenojar, Xinning Wang, Hans Marten Hazelbag, Marieke E. Straver, Okker D. Bijlstra, Taryn L. March, Alexander L. Vahrmeijer, Agata Exner, Matthew Bogyo, James P. Basilion and Brian Straight

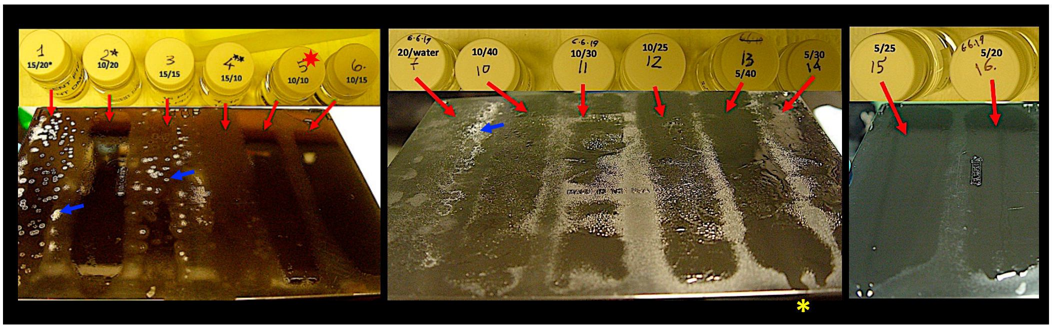

Supplemental Figure S1: Surface behavior of Pluronic F-127 hydrogel / DMSO mix depends on concentration of the Pluronic F-127 (formulations with lower concentrations of the hydrogel). **Formulation:** Percentage of Pluronic F-127 hydrogel and DMSO in the saline-based mixes was as follows: #1 – 15/20, #2 – 10/20, #3 – 15/15, #4 – 15/10, #5 – 10/10, #6 – 10/15, #10 – 10/40, #11 – 10/30, #12 – 10/25, #13 – 5/40, #14 – 5/30, #15 – 5/25, #16 – 5/20, respectively. Mix#7 – gel=20% in water (no DMSO – dimethyl sulfoxide). **Gel application**: four layers of each mix were applied as a strip onto the pre-warmed (37^0C) plastic surface (slope ~20-25^0) by air-gun with a low pressure. Plastic platform was kept at 37^0C for 30-min. **Behavior and Conclusions**: 1) *Yellow asterisks* indicate moving down the slope of mixes #13 and #15 after application; 2) *Blue arrows* – dry spots of the mix#1, #3, and #7 on the surface after application; 3) Mixes #2, #4, #5, #6, #15, and #16 showed smooth distribution and did not dry. However, mixes #15 and #16 appeared to be a too leaky on the surface and mixes #10 – #14 formed a rough granulated film with a tendency to be dried; 4) *Red star* – Gel#5, which was chosen for the topical application of AKRO-QC-ICG probe using an airbrush.

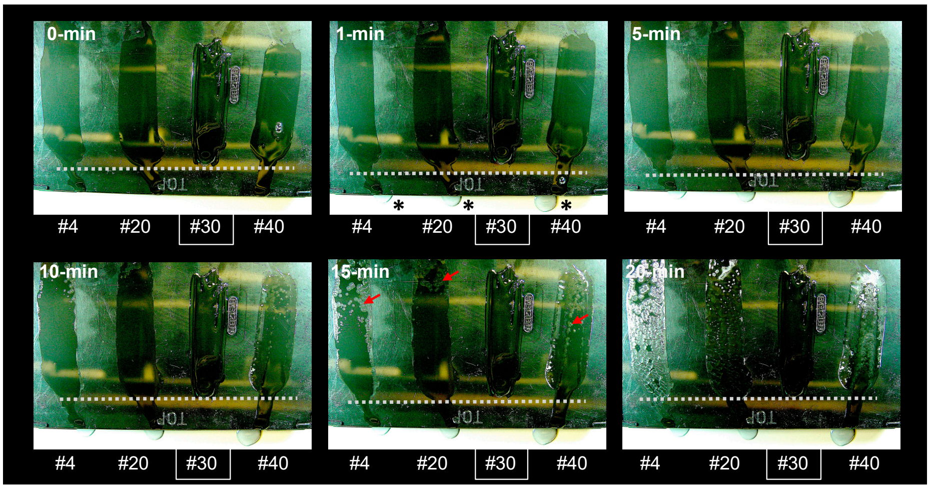

Supplemental Figure S2. Surface behavior of Pluronic F-127 hydrogel / DMSO mix depends on concentration of the Pluronic F-127 (formulations with higher concentrations of the hydrogel). **Formulation:** Percentage of Pluronic F-127 hydrogel and DMSO in the saline-based mixes was as follows: #4 – 15/10, #20 – 10/10, #30 – 17/20, #40 – 15/40, respectively. **Gel application:** Since higher concentration of Pluronic F-127 reduced a fluidity of Pluronic F-127/ DMSO mixes that led to a clogging of airbrush. So, four layers of each mix were applied as a strip onto the pre-warmed (37^0C) plastic surface (slope ~20-25^0) by wood stick cotton swabs . Plastic platform

was kept at 37⁰C for 30-min. **Behavior and Conclusions**: 1) *Black asterisks* indicate moving down the slope of #4, #20, #40 but not #30 mix, 1-min after application that led to a pooling of #4, #20, #40 mixes at the flat desk surface at the bottom; 2) *Red arrows* – dry spots 10-15-min after application; 3) *Dotted grey lines* indicate lower border of applied gel mixes.

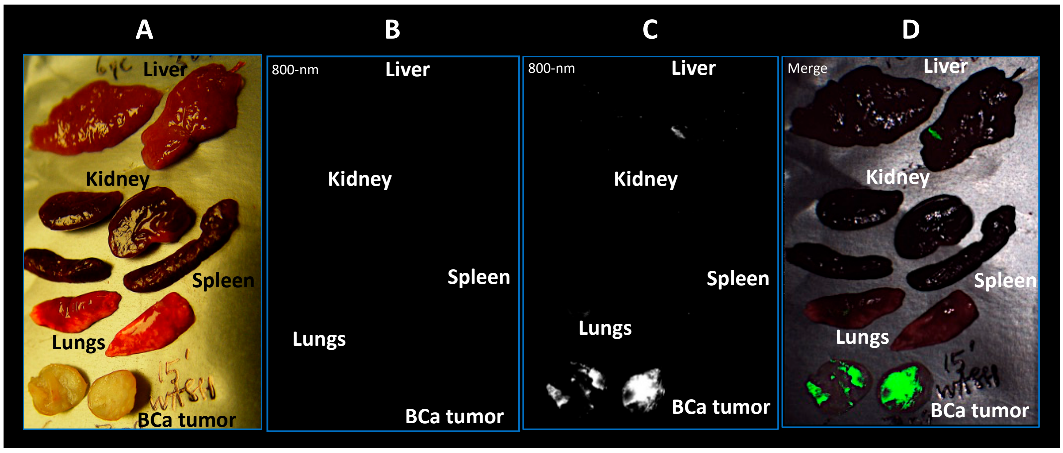

Supplemental Figure S3. AKRO-QC-ICG imaging gel becomes fluorescent (unquenched) only on fresh cuts of the human breast MDA-MB-468 tumor xenograft but not the normal organ tissues after topical application ex vivo. (**A**) – Mouse organs and tumor (color photo) were excised and than cut into 2 halves followed by topical application of AKRO-QC-ICG imaging gels #30 and imaging; **(B)** – ICG fluorescent pre-image of the organs (no probes applied=auto-fluorescence); **(C)** – ICG fluorescent image of the organs and tumor in *(A)*. AKRO-QC-ICG (5-µM final) imaging gel was washed out by sterile saline from all tissue samples after 30-min of topical application followed by imaging; **(D)** – merge of the ICG fluorescence in *(C),* a false green color, and photograph. Camera – Curadel Lab-Flare RP1 with 800-nm filter set. Software – Curadel Resvet Imaging.

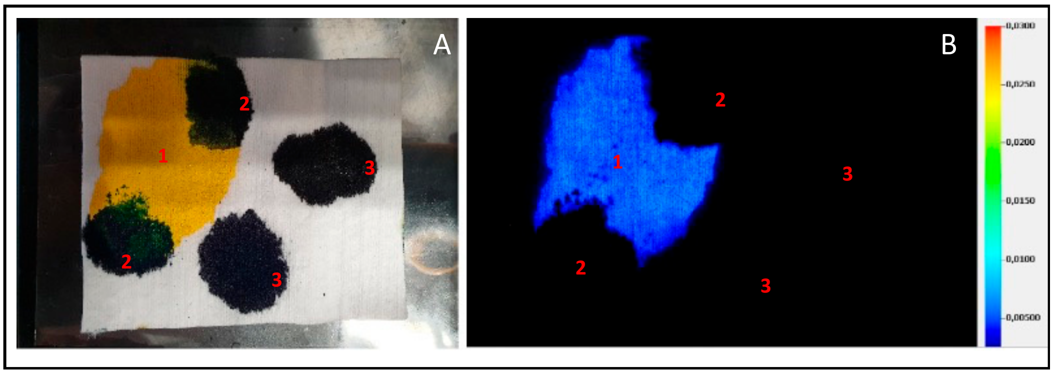

Supplemental Figure S4. Imaging of yellow color pathological ink auto-fluorescence with 800-nm emission filter set. **(A)** – Color photo of yellow (1), green (2), and black (3) color pathology ink spots. **(B)** – Levels of auto-fluorescence of the inks in (A) with ICG-800-nm emission filter set. Camera – Pearl® Trilogy Small Animal Imaging System with 800-nm filter set; software – Image Studio (both Li-Cor Bioscience, NE).

Supplemental Table S1. Clinical and Pathological Characteristics of the Lumpectomy Sample.

Patient	Grade	Clinical stage	Pathological stage	Histology	Receptor status	Ki-67 status	Tumor size
**1**	1	cT1cN0	pT1cN0(i-)(sn)	Invasive carcinoma NST	$ER^+/PR^+/HER2^-$	20%	15-mm

Notes: 1) NST - no special type; 2) ER - estrogen receptor; 3) PR - progesterone receptor; 4) HER2 - human epidermal growth factor receptor-2.
